# Earache

**Published:** 1882-03

**Authors:** Wharton Jones


### EARACHE

"In the course of practice you will often be called upon to attend a case of Earache. This means, pathologically speaking, acute inflammation of the membrana tympani. Now, in such cases you may quickly subdue the inflammation, relieve the patient from the excruciating pain he is suffering, and save him perhaps, from subsequent confirmed deafness. The treatment, from which such a very desirable result may be obtained, is similar to that which you will find so beneficial in analogous cases of eye diseases—viz: leeches behind the ear, hydrag. c. creta and belladonna powders, with warm fomentations."—*Wharton Jones* in *London Lancet.*

